# Leveraging Fitness Tracker and Personalized Exercise Prescription to Promote Breast Cancer Survivors’ Health Outcomes: A Feasibility Study

**DOI:** 10.3390/jcm9061775

**Published:** 2020-06-08

**Authors:** Nan Zeng, Ning Liao, Chunyuan Han, Wenxi Liu, Zan Gao

**Affiliations:** 1 Department of Food Science and Human Nutrition, Colorado State University, Fort Collins, CO 80523, USA; 2 Department of Breast Cancer, Guangdong Academy of Medical Sciences, Guangzhou 510080, China; 3 School of Physical Education, South China University of Technology, Guangzhou 510641, China; chyhan@scut.edu.cn; 4 School of Kinesiology, University of Minnesota, Minneapolis, MN 55455, USA; liux4443@umn.edu

**Keywords:** aerobic endurance, blood glucose, body flexibility, daily steps, wearable technology

## Abstract

Purpose: This feasibility study investigated whether a year-long combined fitness wristband-based and personalized exercise prescription intervention improves Chinese breast cancer survivors’ (BCSs) health outcomes. Methods: Ninety-five BCSs (X_age_ = 44.81 ± 7.94; X_BMI_ = 22.18 ± 3.48) were recruited from Southern region of China and were delivered the exercise intervention across 12 months, using a single group pretest–posttest design. Participants’ lipid profile (e.g., total *cholesterol*, high-density lipoprotein, low-density lipoprotein, and triglycerides), blood glucose, breast cancer biomarkers (e.g., carcinoembryonic antigen and cancer antigen 15-3), and functional fitness (e.g., strength in arms and legs, endurance, balance, agility, and flexibility) were assessed at baseline and 12-month post-intervention. Results: Thirty-three BCSs successfully completed the intervention. A significant change in blood glucose (mean difference (MD): −0.22, 95% confidence interval (CI): −0.41–−0.03, *t* = −2.25, *p* = 0.028) was observed, with participants demonstrating lower levels of blood glucose at the 12-month post-intervention versus the baseline assessment. Notable changes in functional fitness were also discerned, including agility and balance (MD: −0.47, 95% CI: −0.68–−0.26, *t* = −4.336, *p* < 0.001), aerobic endurance (MD: 89.25, 95% CI: 73.82–104.68, *t* = 11.336, *p* < 0.001), lower-body flexibility (left) (MD: 4.58, 95% CI: −4.4–13.56, *t* = 4.653, *p* < 0.001), and lower-body flexibility (right) (MD: 4.84, 95% CI: −4.65–14.33, *t* = 4.092, *p* < 0.001). Conclusion: The observations suggested that our behavioral change program might promote certain health outcomes in Chinese BCSs, yet we are unable to recommend such a program given existing limitations. Future randomized control trials with diverse samples are warranted to confirm our findings.

## 1. Introduction

The most recent global estimates suggested that about 1.7 million new cases of breast cancer are diagnosed every year worldwide and that the vast majority of whom are females [1]. Cancer brings patients with physical, physiological, and psychological side effects including muscular atrophy, weight changes, lowered aerobic capacity, decreased strength and flexibility, fatigue, depression, and an overall decline in the quality of life [2]. Due to treatment advancements, luckily, survival following breast cancer diagnosis has greatly increased, with the average five-year net survival for women having invasive breast cancer remaining highest in developed countries such as U.S. (90%) [3] and Canada (87%) [4]. In contrast, however, approximately 60% of deaths from breast cancer occurred in developing countries [1], representing a huge variation among countries. In China, the five-year survival rate for breast cancer patients has reached 83.2%, which illustrated a 7.3% increase over the past decade [5]. However, the five-year survival rate is still lower than that of developed countries. 

Clinical practice guidelines recommend that breast cancer survivors (BCSs) should receive health promotion counselling in relation to obesity, physical activity (PA), nutrition, and smoking cessation [6,7,8]. Supported by scientific evidence, major health organizations such as the U.S. Department of Health and Human Services, the American Cancer Society, and the *American College of Sports Medicine* (ACSM) commonly identify the importance of incorporating PA into cancer care and advise patients to be physically active [9,10]. Review evidence also demonstrated the beneficial role of PA in mitigating several adverse effects of breast cancer and its treatment, including fatigue [11], upper-limb dysfunction [12], PA level, aerobic fitness, muscular strength, anxiety, self-esteem, and quality of life [13]. A recent systematic review indicated that patients who exercised following a diagnosis of cancer tended to have a lower relative risk of cancer mortality and recurrence and to experience fewer/less severe adverse effects [14], echoing the view that PA has been an important holistic therapy for the disease management in BCSs. 

BCSs’ PA levels decrease remarkably after breast cancer diagnosis and increase only slowly after the treatment period, causing a decline in functional fitness and overall well-being [15,16]. Despite successfully promoting people’s knowledge and favorable intentions to adopt healthy behaviors, interventions to change health behaviors have largely failed to establish enduring healthy lifestyle habits due to insufficient intervention lengths [17], highlighting that long-term behavior change is the key to developing healthy habits. This corresponds with previous research that the short-term PA programs would likely limit the ability to detect specific physiological responses with interventions in cancer patients [18], and thus, larger trials that examine the long-term benefits of PA are needed for this patient group [19].

While evidence for the benefits of PA in BCSs continues to grow, the application of well-established exercise-training principles in the field of PA research and practice are not commonly being reported in the oncology literature [20]. Incomplete reporting of the exercise prescription (i.e., components of frequency, intensity, time, and type) and adherence to the prescription would restrict the reproducibility of PA intervention and the ability to determine the PA dose received by BCSs. This mirrors most review evidence that no PA studies in women diagnosed with breast cancer (1) attend to all key principles of exercise training, (2) describe all components of the exercise prescription in the methods, and (3) report adherence to the prescribed intervention in the results [21], all of which underline the need for reliable clinical trials that focus on delivering evidence-based, elaborate, and personalized exercise prescriptions. 

Today, the application of emerging technology in health promotion has generated substantial public interest [22], with wearable technology such as smartwatches and fitness wristbands being the most exciting and technologically advanced. This is because of its ability to track health metrics like step counts, heart rate, energy expenditure, and sleep [23]. Applying emerging technology to encourage PA among various populations, therefore, has generated substantial public interest [22]. It is worth noting that wearable activity trackers are perceived as useful and acceptable tools by BCSs for self-regulation of PA and sedentary behaviors and thus are recommended for health promotion [24]. Previous studies have also observed wearable devices such as Polar smartwatch to be effective in promoting PA, quality of life, and healthy weight status in BCSs [25,26], yet little to no research has been conducted on the long-term effectiveness of wearable activity trackers in the promotion of this population’s physical and clinical outcomes. It is also suggested that interventions may be more effective if focused on culturally appealing personalized exercise behaviors [27]. Therefore, we conducted the current study to investigate the feasibility of using a commercially available fitness tracker, employed in conjunction with a personalized exercise prescription to promote BCSs’ health outcomes.

## 2. Methods

### 2.1. Participants

BCSs were recruited via posted flyers and word of mouth in Guangdong Provincial People’s Hospital. Interested BCSs were thereafter contacted by the researcher staff for screening against the following inclusion criteria: (1) previously diagnosed with stages 0–III breast cancer; (2) completed primary cancer treatment between 1–5 years earlier with no new cancer diagnosis or recurrence; and (3) did not participate in other health promotion programs. Exclusion criteria included (1) currently undergoing breast cancer treatment and (2) having any contraindications that might interfere with PA engagement, such as medical conditions and pacemaker implant. 

### 2.2. Study Design and Procedure 

The current study was conducted between April 2016 and July 2017 in the city of Guangzhou, a tier-one metropolitan area in Southern China, using a single group pretest-posttest design. Specifically, BCSs were recruited from early April to mid-June 2016, with those who were interested in participating contacted by the research staff and screened against inclusion criteria. During this period, the baseline assessment was conducted with the eligible BCSs, including demographic/anthropometric information, clinical characteristics, and health outcomes. Notably, ethnicity and socioeconomic status were not used as enrollment screening criteria and thus were not collected in the current study. The BCSs were then provided with a Xiaomi wristband (Mi Band 1S Heart Rate Wristband, Xiaomi Corporation, Beijing, China) to monitor activity behaviors for one month at the end of June 2016. Like other fitness trackers (e.g., Fitbit Charge 3 and Polar A370), the Mi band can track health metrics, including steps taken, heart rate, calories burned, etc. In comparison to other commercially available fitness trackers, however, the Mi band has the advantage of lower price, which only costs between $20 to $25 *USD*. At the end of July 2016, all BCSs were instructed via phone to upload their health metrics data on *the Mi band* to a shared online server provided by the research team with the assistance of a high-tech company.

It has been recommended that sedentary women should take 10,000 steps per day to receive health benefits [28,29]. Therefore, 10,000 steps per day was set as the median cutoff points for the 36 step categories while clustering BCSs with specific exercise prescriptions (see Table 1). Based upon daily steps taken for the past month, BCSs were first categorized into groups: (1) ≤7999 steps/day, (2) 8000–12,799 steps/day, and (3) ≥12,800 steps/day. In each category, BCSs were further classified as 12 different levels of exercise prescription. For example, if one was categorized as group 1 (e.g., ≤7999 steps/day) and her actual daily steps taken were less than 2499, she was then provided the level 1.1 exercise prescription. If one’s daily steps were 2500–2999, then that patient was given the level 1.2 exercise prescription (see Appendix A). The specific classification is presented in Table 1. To initiate the intervention, a personalized exercise prescription for next month (4 weeks) was delivered to each BCS by the research staff at the end of July 2016. For instance, BCSs given the level 1.1 exercise prescription were instructed to perform 10-min strengthening (e.g., squat and bicycle crunch) and 30-min moderate aerobic exercise (e.g., brisk walk) on day 1 and 10-min strengthening on different muscle groups and 30-min moderate aerobic exercise on day 2. Some sample specific exercise prescriptions for several levels (i.e., 1.1, 1.2, 2.1, and 2.2) are described in Appendix A, which were guided by the ACSM Exercise Guidelines for Cancer Survivors [9].

To ensure that our health behavior change program worked while avoiding other confounders, BCSs were instructed not to participate in other health promotion programs for the duration of the study. During the 12-month intervention, BCSs were asked not to change their usual diet and were required to implement their own exercise prescriptions weekly and to wear the Mi band to track their steps taken daily. The activity data (i.e., average daily steps taken) collected by the Mi band for the past month were manually uploaded to an online server and received by the research staff at the end of each month. Afterwards, the personalized exercise prescriptions for the next month based on steps were delivered to BCSs. The intervention lasted 12 months until July 2017. Post-intervention assessments including all outcomes outlined previously were conducted at the end of July *2017*. All BCSs received the Mi band as the nonmonetary incentive, regardless of whether they successfully completed all study procedures or not. All procedures performed with BCSs in the current study were in accordance with the ethical standards of the institutional and/or national research committee and with the 1964 Helsinki declaration and its later amendments or comparable ethical standards [30]. University approval and informed consent were documented prior to any data collection.

### 2.3. Measurements

*Demographic and Anthropometric Information:* BCSs self-reported date of birth, breast cancer diagnosis stage, menstrual status, and types of breast cancer. Height without shoes was measured to the nearest 0.1 cm using a Seca stadiometer (Seca, Hamburg, Germany) while weight and body fat percentage were assessed via the Tanita BC-558 IRONMAN^®^ Segmental Body Composition Monitor (Tanita, Tokyo, Japan) after voiding or wearing light clothing and no shoes or socks. Body mass index (BMI) was defined as weight/height^2^ and was expressed in kilograms per square meter. Waist circumference was measured to the nearest 0.1 cm using a retractable medical tape in the standing position at the level of the umbilicus, and hip circumference was measured to the nearest 0.1 cm at the largest posterior extension of the buttocks. 

*Health Outcomes*. All testing was conducted at Guangdong Provincial People’s Hospital, including lipid profile (i.e., total cholesterol, high-density lipoprotein, low-density lipoprotein, and triglycerides), blood glucose, breast cancer biomarkers (i.e., carcinoembryonic antigen and cancer antigen 15-3), and functional fitness (i.e., strength in arms and legs, endurance, balance, agility, and flexibility) were assessed at baseline and 12-month post-intervention. Specifically, fasting venous blood samples of the participants were drawn and analyzed by the ADVIA 2400 Chemistry System (Siemens Healthcare Diagnostics) for the measurements of total cholesterol, high- and low-density lipoprotein, triglycerides, and blood glucose. In addition, serum levels of carcinoembryonic antigen and cancer antigen 15-3 were determined via electrochemiluminescence immunoassay and chemiluminescence enzyme immunoassay, using a kit provided by Roche Diagnostics Ltd. (Rotkreuz, Switzerland) and Fujirebio Inc. (Tokyo, Japan), respectively. To ensure the precision and accuracy of measurement, daily internal quality control was performed with commercial quality control materials (BioRad Labs). Patients’ functional fitness was assessed via the Senior Fitness Test (SFT), which consists of six functional measures of strength in arms and legs, endurance, balance, agility, and flexibility [31]. The detailed description of the SFT is presented in Table 2. The SFT battery has been shown high reliability and validity (intraclass correlation coefficient (ICC) ranged between 0.79 and 0.97) in assessing physical functioning [32] and has been widely used among various clinical populations, including BCSs [33]. The SFT battery requires approximately 30–40 min per patient to administer. 

### 2.4. Statistical Analysis

All analyses were conducted in the open-access software RStudio version 3.5.0 (The R Foundation, Vienna, Austria). Data were first screened for the outliers and normality of distributions, after which the outliers were adjusted to lessen the impact of extreme scores and the skewed data were log transformed to improve shape characteristics. Second, descriptive statistics were calculated and presented as mean ± standard deviation and as frequency, unless stated otherwise. Lastly, to evaluate significant changes on BCSs’ health outcomes, linear mixed models were performed, with the participant being treated as the random effect. This analytic approach reduces concerns regarding missing data, as it allows to incorporate all available data and takes into account the fact that participants were lost during the study period due to withdrawal or loss of contact [34]. All models were fitted using the lme4 package [35], and the t-tests used in the interpretation of model effects were computed using the ImerTest package [36]. Statistical significance was set at *p* < 0.05. Notably, sensitivity analysis was also performed using only the complete-case data and showed similar results. 

## 3. Results

A total of 175 BCSs were screened for eligibility, with 80 who failed to meet inclusion criteria were excluded. As a result, 95 BCSs (X_age_ = 44.81 ± 7.94; X_BMI_ = 22.18 ± 3.48) initially enrolled and started the intervention. Notably, 62 BCSs were considered incomplete cases due to (1) inadequate adherence (defined as less than 50% of prescribed sessions attended) to the prescribed intervention; (2) inability to finish the post-assessment; (3) changes in health status unrelated to the study and thus cessation of the intervention; (4) withdrawal; and (5) loss of contact. Finally, a total of 33 BCSs successfully finished all the study protocols and had complete pre- and post-assessment data, causing a retention rate of 35%. Complete baseline characteristics of the samples, including initial, nonadherence, and adherence are presented in Table 3. Despite low retention, a good to excellent adherence to the exercise prescription was self-reported by these participants, with adherence rate ranging from 75% to 90%, and the mean adherence rate was 83%. Of note, we also examined the baseline differences in participants characteristics between nonadherence and adherence samples and found no significant difference between these two subgroups (data are not shown).

Descriptive characteristics for the outcomes of interest at baseline and 12-month as well as inferential statistics for baseline and posttest on health outcomes are shown in Table 4. Specifically, there was a significant change in blood glucose over time (mean difference (MD): −0.22, 95% confidence interval (CI): −0.41–−0.03, *t* = −2.25, *p* = 0.028), with BCSs demonstrating lower levels of blood glucose at the 12-month versus baseline assessment. Significant changes on other health outcomes were not observed, including cancer antigen 15-3 (MD: 0.006, 95% CI: −1.394–1.406, *t* = 0.008, *p* = 0.993); carcinoembryonic antigen (MD: 0.13, 95% CI: −0.12–0.38, *t* = 0.301, *p* = 0.759); high-density lipoprotein (MD: −0.05, 95% CI: −0.14–0.04, *t* = −0.929, *p* = 0.362); low-density lipoprotein (MD: −0.03, 95% CI: −0.10–0.04, *t* = −0.230, *p* = 0.820); total cholesterol (MD: −0.14, 95% CI: −0.42–0.14, *t* = −0.754, *p* = 0.456)*;* and triglyceride (MD: 0.18, 95% CI: −0.18–0.54, *t* = 0.916, *p* = 0.364). 

**Figure 1:**
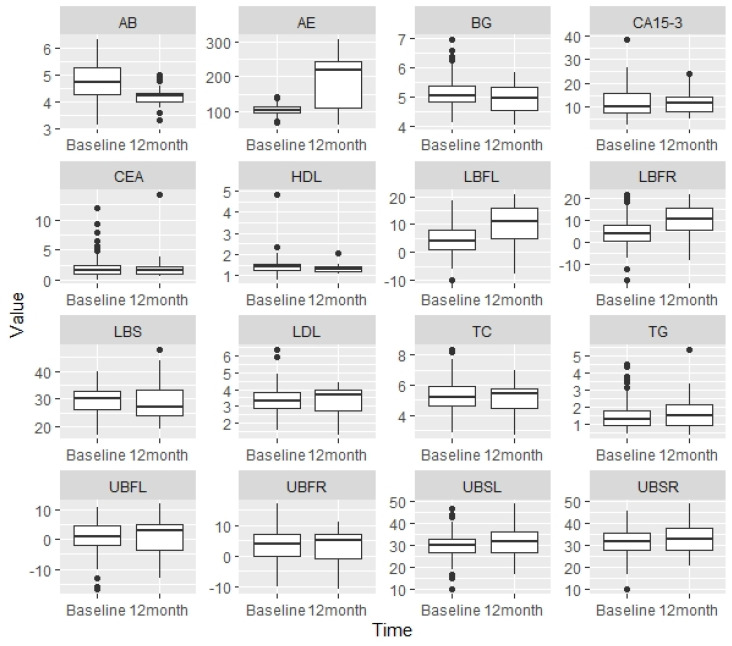
Comparisons between baseline and 12 months across all outcomes of interest. Note: AB = agility and balance; AE = aerobic endurance; BG = blood glucose; CA15-3 = cancer antigen 15-3; CEA = carcinoembryonic antigen; HDL = high-density lipoprotein; LBFL = lower-body flexibility left; LBFR = lower-body flexibility right; LDL = low-density lipoprotein; LBS = lower-body strength; UBFL = upper-body flexibility left; UBFR = upper-body flexibility right; TC = total cholesterol; TG = triglyceride; UBSL = upper-body strength left; UBSR = upper-body strength right.

The results also suggested that our behavior change program was effective at improving BCSs’ functional fitness, including agility and balance (MD: −0.47, 95% CI: −0.68–−0.26, *t* = −4.336, *p* < 0.001), aerobic endurance (MD: 89.25, 95% CI: 73.82–104.68, *t* = 11.336, *p* < 0.001), lower-body flexibility (left) (MD: 4.58, 95% CI: −4.4–13.56, *t* = 4.653, *p* < 0.001), and lower-body flexibility (right) (MD: 4.84, 95% CI: −4.65–14.33, *t* = 4.092, *p* < 0.001). Of note, BCSs’ aerobic endurance demonstrated the greatest improvements among all outcomes after the 12-month behavior change program. No significant changes were detected in lower-body strength (MD: −0.47, 95% CI: −1.39–0.45, *t* = −0.486, *p* = 0.629), upper-body flexibility (left) (MD: 1.40, 95% CI: −1.34–4.41, *t* = 1.632, *p* = 0.110), upper-body flexibility (right) (MD: 0.43, 95% CI: −0.41–1.27, *t* = 0.782, *p* = 0.439), upper-body strength (left) (MD: 2.31, 95% CI: −2.21–6.83, *t* = 1.983, *p* = 0.053), and upper-body strength (right) (MD: 1.70, 95% CI: −1.64–5.04, *t* = 1.456, *p* = 0.151). Visual comparisons between the baseline and 12 months across all outcomes of interest are exhibited in Figure 1. 

## 4. Discussion

BCSs are at risk of greater physical inactivity and sedentary behavior [37]. PA engagement after a breast cancer diagnosis may reduce the risk of death from this disease, with patients who follow PA guidelines are more likely to improve their survival [38]. Previous research suggested that the personalized interventions are effective in promoting healthful lifestyle changes among cancer survivors [39]. Additionally, BCSs have found acceptable and expressed interest in the use of wearable activity trackers to self-regulate their activity behaviors [24]. Therefore, this study examined the feasibility of a combined wristband-based and personalized exercise prescription intervention in the promotion of improved health outcomes in Chinese BCSs. Our observations demonstrated that this type of intervention might improve patients’ blood glucose levels, agility and balance, aerobic endurance, and lower-body flexibility. 

Physiological and biological indicators play an important role in the management of BCSs [40] and provide a guide for the treatment of breast cancer. Review evidence from randomized controlled trials (RCTs) that included clinical endpoints demonstrated that exercise may trigger beneficial changes in circulating insulin level, insulin-related pathways, inflammation, serum level of high-mobility group box 1, serum interleukin 6 and tumor necrosis factor-*α*, leptin, total cholesterol, cutaneous T cell-attracting chemokine, and possibly immunity [41,42,43]. Notably, while the positive changes after PA intervention are promising, these changes were not consistently and statistically significant across all included studies, suggesting that the current evidence concerning the effects of exercise on biomarkers in BCSs is controversial. 

Our observations were congruent with this assertion; only detected blood glucose was significantly improved following a 12-month combined aerobic and resistance training intervention, among other health outcomes. Interestingly, a recent study (Breast Cancer and Exercise Trial in Alberta) [44] by Friedenreich et al. also examined the effects of a year-long PA intervention on BCSs’ biomarkers, including C-reactive protein, insulin, glucose, homeostatic model assessment of insulin resistance, estrone, sex hormone-binding globulin, total estradiol, and free estradiol. In their study, the 400 BCSs were randomly assigned to either moderate-vigorous aerobic exercise, 5 days/week (3 days/week supervised) for 30 min/session (moderate), or 60 min/session (high) conditions. Corresponding with our findings, this study observed that some biomarkers improved for both groups after one year but that neither group observed a significantly greater improvement over the other. This signifies that performing either moderate-duration or high-duration exercises can reduce the risk of breast cancer. It is noteworthy, however, in their study that some biomarkers such as estrogen and insulin stayed the same over time because they are already low in postmenopausal women.

It is suggested that PA may be more pronounced on certain biomarkers (e.g., insulin pathway) in obese or physically inactive BCSs [41,45]. Notably, BCSs in our study were relatively fit and active; this could partially explain why other biomarkers were not found to be improved. Moreover, there was also a suggestion that PA may be more effective at modifying certain biomarkers such as insulin-like growth factor 1 in BCSs who were not taking tamoxifen [41]. However, in the present study, we did not investigate whether and how many BCSs were taking tamoxifen during the intervention period, which, to some extent, might be a confounding factor that impacts our experimental results. In closing, while our study observed that a year-long PA program may improve BCSs’ blood glucose levels, future research is warranted to confirm the effects of this innovative intervention on multiple biomarkers.

Breast cancer and oncologic treatment may generate significant negative effects on physical functioning [46]. Women who have been diagnosed with breast cancer often experience a rapid decline in functional fitness [47]. The American Cancer Society recommends that BCSs should engage in PA regularly to improve their functional fitness and to reduce the risk of developing new cancers [48]. Evidence consistently demonstrated that PA led to significant improvements in aerobic fitness, increased flexibility, and strength [9]. For example, Milne et al. [49] conducted an RCT of combined aerobic and resistance exercise in BCSs over 24 weeks and observed significant improvements in functional fitness, including aerobic fitness (i.e., the Aerobic Power Index cycle test) and muscular strength (i.e., bicep curls, leg press, and chest extension). Likewise, another larger RCT by Winters-Stone et al. [50] found that BCSs in the resistance training group had better testing scores on maximal leg and bench press strength compared to the stretching group over the course of a year-long intervention, despite no significant improvements noted in grip strength, chair stand, best 4-m usual walk, and one-leg standing balance tests. 

Moreover, Foley and Hasson [51] recently conducted a 12-week (30 min each, totaling 90 min twice weekly) multimodal exercise intervention (a combination of aerobic conditioning, resistance, balance, and flexibility training) using one single-group pretest–posttest design, to promote BCSs’ functional fitness. The study observed significant improvements in mobility (Timed Up and Go, 6-min walk test), muscular strength (leg press strength and chest press strength), upper-extremity flexibility (back scratch test), and balance (functional reach test and single-leg stance time). Interestingly, our study only observed notable changes in agility and balance, aerobic endurance, and lower-body flexibility but not in lower-body strength, upper-body flexibility, and upper-body strength. It is noteworthy, however, that our sample was recruited from China, whereas Foley and Hasson’s sample was from North America. Previous research appealed that multilevel analysis of individuals and populations within specific contexts would strengthen clinical practice for cancer management [52,53]. Therefore, future studies with racially and ethnically diverse groups are encouraged to ascertain such disparities. The widening disparities in cancer outcomes between Asian- and Euro-Americans challenges the current research and practice paradigms for cancer control. A Cultural Systems Approach would strengthen future studies. This paradigm requires multi-level analyses of individuals and populations within specific contexts in order to identify culturally based strategies to improve practice along the cancer care continuum.

The strengths of this study include (1) the application of a combined wristband-based and personalized exercise prescription intervention in BCSs, making it one of its first kind in the literature while providing the preliminary evidence for the acceptability and feasibility of this type of innovative intervention; (2) all components of the customized exercise prescription are driven by clinical practice guidelines, warranting the tolerability and safety of the intervention; and (3) detailed reporting of both exercise prescription and adherence to exercise prescription, which well responds to the call to action [21] and would allow full replication of positive findings in clinical settings. Several limitations within this study, however, should also be noted. First, this study was conducted in one geographic location in a sample of Chinese BCSs, which limits generalizability of observations and somewhat hinders the ability to identify racial differences. Second, we only studied a single group of BCSs and did not include a control group, impeding any ability to draw initial intervention effectiveness conclusions. Third, the study implementation was limited by a high attrition rate that may increase the risk of bias. Indeed, it must be admitted that the retention strategies in this study may not be efficient, which resulted in a high attrition rate while increasing the risk of bias. Nevertheless, clinically promising trends in certain biomarkers and functional fitness have been noted. Fourth, as a feasibility study, we failed to collect patients’ past treatment exposures, which can be confounders and may potentially influence our observations. Future studies are encouraged to expand on this effort. Fifth, due to our recruitment method, differences in physical fitness may preexist among BCSs and thus may potentially impact all outcome measures. Finally, and relatedly, due to low retention rate, qualitative approach was not undertaken, which limits the ability to optimize our design. 

## 5. Conclusions

This study provides the preliminary evidence that a year-long combined fitness wristband-based and individually tailored aerobic and resistance training might be able to promote improved blood glucose levels, agility and balance, aerobic endurance, and lower-body flexibility in Chinese BCSs. However, our observations do not suggest that the programming confers additional beneficial effects on other health outcomes. Given that the findings of this feasibility study were related to the preceding limitations, we are unable to recommend implementing such behavior change program in BCSs yet. Future studies addressing the above limitations with more rigorous study design (e.g., RCTs with mixed methods), racially and ethnically diverse samples, as well as multiple health assessments are warranted to confirm and generalize our findings. 

## Figures and Tables

**Table 1:** Categories of exercise prescription.

Category	Average Steps/Day	Exercise Prescription
≤7999 steps/day	≤2499 steps/day	Level 1.1
	2500–2999 steps/day	Level 1.2
	3000–3499 steps/day	Level 1.3
	3500–3999 steps/day	Level 1.4
	4000–4499 steps/day	Level 1.5
	4500–4999 steps/day	Level 1.6
	5000–5499 steps/day	Level 1.7
	5500–5999 steps/day	Level 1.8
	6000–6499 steps/day	Level 1.9
	6500–6999 steps/day	Level 1.10
	7000–7499 steps/day	Level 1.11
	7500–7999 steps/day	Level 1.12
8000–12,799 steps/day	8000–8399 steps/day	Level 2.1
	8400–8799 steps/day	Level 2.2
	8800–9199 steps/day	Level 2.3
	9200–9599 steps/day	Level 2.4
	9600–9999 steps/day	Level 2.5
	10,000–10,399 steps/day	Level 2.6
	10,400–10,799 steps/day	Level 2.7
	10,800–11,199 steps/day	Level 2.8
	11,200–11,599 steps/day	Level 2.9
	11,600–11,999 steps/day	Level 2.10
	12,000–12,399 steps/day	Level 2.11
	12,400–12,799 steps/day	Level 2.12
≥12,800 steps/day	12,800–13,099 steps/day	Level 3.1
	13,100–13,399 steps/day	Level 3.2
	13,400–13,699 steps/day	Level 3.3
	13,700–13,999 steps/day	Level 3.4
	14,000–14,299 steps/day	Level 3.5
	14,300–14,599 steps/day	Level 3.6
	14,600–14,899 steps/day	Level 3.7
	14,900–15,199 steps/day	Level 3.8
	15,200–15,499 steps/day	Level 3.9
	15,500–15,799 steps/day	Level 3.10
	15,800–16,099 steps/day	Level 3.11
	≥16,100 steps/day	Level 3.12

**Table 2:** Description of senior fitness test.

Item	Description
Chair Stand	The number of full stands with arms folded across the chest completed in 30 s was used to represent lower-body strength.
Arm Curl	The number of *5*-pound (2.28 kg) dumbbell biceps curls completed in 30 s were used to represent upper-body strength.
Chair Sit-and-Reach	From a sitting position at the front of chair with leg extended and hands reaching toward toes, the number of centimeters between extended fingers and tip of toe (+/−) was used to represent lower-body flexibility.
Back Scratch	With one hand reaching over the shoulder and one up the middle of the back, the number of centimeters between extended middle fingers (+/−) was used to represent upper-body flexibility.
8-Foot Up-and-Go	The number of seconds required to get up from a seated position, to walk 8 feet (2.44 m), to turn, and to return to a seated position were used to represent agility and balance.
2-Minute Step	The number of times that the right knee reaches the tape level (midway between the patella and iliac crest in 2 min were used to represent aerobic endurance.

**Table 3:** Participant baseline characteristics.

Characteristics	Initial Sample (*N* = 95)	Nonadherence (*N* = 62)	Adherence (*N* = 33)
^a^ Age (year)	44.81 ± 7.94	44.98 ± 8.48	44.48 ± 6.93
^a^ Height (cm)	158.18 ± 4.39	158.14 ± 4.74	158.27 ± 3.74
^a^ Weight (kg)	55.21 ± 7.69	55.22 ± 8.28	55.19 ± 6.59
^a^ Body Mass Index (kg/m^2^)	22.18 ± 3.48	22.27 ± 3.84	21.99 ± 2.73
^a^ Waist Circumference (cm)	77.68 ± 9.26	77.17 ± 10.06	78.64 ± 7.57
^a^ Hip Circumference (cm)	93.47 ± 6.57	93.77 ± 6.54	92.91 ± 6.70
^b^ Diagnosed breast cancer stage			
Stage 0	5	3	2
Stage I	26	17	9
Stage IIa	38	26	12
Stage IIb	14	11	3
Stage IIIa	10	3	7
Stage IIIc	2	2	0
^b^ Menstrual status			
Postmenopause	52	32	20
Premenopause	43	27	16

Note: ^a^ Data are presented as mean ± standard deviation; ^b^ data are presented as frequency; no statistically significant difference *in baseline* characteristics between nonadherence and adherence.

**Table 4:** Inferential statistics for baseline and posttest on health outcomes.

Health Outcomes	Baseline (*N* = 95)	12-Month (*N* = 33)	Estimate (95% CI)	*p* Value
Blood Glucose (mmol/L)	5.14 ± 0.47	4.92 ± 0.52	−0.22 (−0.41–−0.03)	0.028 *
Cancer Antigen 15-3 (U/mL)	11.94 ± 5.71	12.01 ± 5.19	0.006 (−1.394–1.406)	0.993
Carcinoembryonic Antigen (ng/mL)	2.15 ± 1.88	2.26 ± 2.57	0.13 (−0.12–0.38)	0.759
High-Density Lipoprotein (mmol/L)	1.46 ± 0.45	1.33 ± 0.21	−0.05 (−0.14–0.04)	0.362
Low-Density Lipoprotein (mmol/L)	3.35 ± 0.90	3.35 ± 0.85	−0.03 (−0.10–0.04)	0.820
Total Cholesterol (mmol/L)	5.29 ± 1.07	5.19 ± 1.05	−0.14 (−0.42–0.14)	0.456
Triglyceride (mmol/L)	1.51 ± 0.85	1.71 ± 1.11	0.18 (−0.18–0.54)	0.364
Functional Fitness				
Agility and Balance (seconds)	4.76 ± 0.07	4.22 ± 0.34	−0.47 (−0.68–−0.26)	<0.001 *
Aerobic Endurance (times)	105.39 ± 14.20	194.64 ± 70.25	89.25 (73.82–104.68)	<0.001 *
Left Lower-Body Flexibility (cm)	4.93 ± 5.59	10.37 ± 6.96	4.58 (−4.4–13.56)	<0.001 *
Right Lower-Body Flexibility (cm)	4.98 ± 6.83	10.73 ± 6.62	4.84 (−4.65–14.33)	<0.001*
Lower-Body Strength (times)	28.98 ± 5.08	28.58 ± 6.88	−0.47 (−1.39–0.45)	0.629
Left Upper-Body Flexibility (cm)	0.61 ± 5.68	1.11 ± 5.92	1.40 (−1.34–4.41)	0.110
Right Upper-Body Flexibility (cm)	3.05 ± 5.38	3.10 ± 5.92	0.43 (−0.41–1.27)	0.439
Left Upper-Body Strength (times)	29.44 ± 6.67	31.84 ± 7.11	2.31 (−2.21–6.83)	0.053
Right Upper-Body Strength (times)	31.93 ± 6.45	34.00 ± 6.87	1.70 (−1.64–5.04)	0.151

Note: All values are presented as mean ± standard deviation; estimates were determined from unadjusted mean differences in group change from baseline to 12 months; 95% CI = 95% confidence interval; * statistical significance within-group changes between baseline and 12 months.

## Appendix A.

(*Selected* exercise prescriptions)

Level 1. Exercise Made Easy: Beginners Programming

Below is a 12-week outline (48 workouts) of exercise programming for individuals with little experience in the gym or those just coming back from a long time away from the gym. As such, this workout program is programmed for exercise 4 times a week for roughly an hour, with simple exercises placed within. Notably, under each of the main exercises for each day are suggested alternative exercises which may better appeal to breast cancer survivors recovering from treatment.

Finally, for each cardio workout listed below, you will use the Borg Rating of Perceived Exertion scale to gauge your intensity level. This scale is listed below, allowing for comprehension of the intensity level; each cardio workout should be completed at.

Month 1: Endurance Phase

For each strength exercise, the endurance phase of this program will include higher numbers of repetitions (10–12 repetitions for each set) for each exercise with lower weight. You should rest for 60 s to 1.5 min between each strength exercise. For each cardio workout, feel free to select your favorite cardio exercise. Some cardio exercise options include but are not limited to walking, jogging, running, biking, swimming, stairclimbing, rowing, elliptical exercise, etc.

### Appendix A.1. Week 1

Day 1:

- Strength Workout
  ○ Dumbbell Lunges (count every other leg): 3 sets × 10–12 repetitions
    ■ Alternative: Walking Lunges without Dumbbells (still counts every other leg)
  ○ Hamstring Curls: 3 sets × 10–12 repetitions
    ■ Alternative: Glute Kickbacks
  ○ Calf Raises: 3 sets × 50 repetitions
    ■ Alternative: Seated Heel Lifts
  ○ Bicycle Crunches: 2 sets × 50 revolutions
    ■ Alternative: Standard Crunches
- Cardio Workout
  ○ 30-min selected cardio exercise at moderate pace: rating of perceived exertion (RPE) Scale: 4–6

Day 2:

- Strength Workout
  ○ Dumbbell Bench Press: 3 sets × 10–12 repetitions
    ■ Alternative: Push-Ups on Knees (only go down as far as your mobility will allow)
  ○ Tricep Kickbacks: 3 sets × 10–12 repetitions
    ■ Alternative: Tricep Dips on a Chair (only go down as far as your mobility will allow)
  ○ Dumbbell Chest Flies (go light and pretend to “hug a tree”): 3 sets × 10–12 repetitions
    ■ Alternative: Wide-Grip Wall Push-Ups
  ○ Bicycle Crunches: 2 sets × 50 revolutions
    ■ Alternative: Standard Crunches
- Cardio Workout
  ○ 30-min selected cardio exercise at high-intensity intervals: These intervals will be comprised of 1 min easy (RPE Scale: 2–3) and 1 min hard (RPE Scale: 7–8) and will be repeated until time is up.

Day 3:

- Strength Workout
  ○ Upright Rows: 3 sets × 10–12 repetitions
    ■ Alternative: Forward Arm Raises
  ○ One-Arm Rows (pretend you are “starting a lawnmower”): 3 sets × 10–12 repetitions
    ■ Alternative: Single Arm Circles
  ○ Lat Pulldowns: 3 sets × 10–12 repetitions
    ■ Alternative: Seated Reaching Exercise (count every other side)
  ○ Dumbbell Shrugs: 2 sets × 10–12 repetitions
    ■ Alternative: Shoulders to Ears
  ○ Sit-ups: 2 sets × 25 repetitions
    ■ Alternative: Standard Crunches
- Cardio Workout
  ○ 30-min selected cardio exercise at moderate pace: RPE Scale: 4–6

Day 4:

- Cardio Workout Only
  ○ 60-min selected cardio at light/moderate pace: RPE Scale: 3–6

### Appendix A.2. Week 2

Day 5:

- Strength Workout
  ○ Goblet Squats with Dumbbell: 3 sets × 10–12 repetitions
    ■ Alternative: Air Squats
  ○ Hamstring Curls: 3 sets × 10–12 repetitions
    ■ Alternative: Glute Kickbacks
  ○ Calf Raises: 3 sets × 50 repetitions
    ■ Alternative: Seated Heel Lifts
  ○ Leg Raises: 2 sets × 25 raises
    ■ Alternative: Seated Leg Lifts
- Cardio Workout
  ○ 30-min selected cardio exercise at moderate pace: RPE Scale: 4–6

Day 6:

- Strength Workout
  ○ Incline Dumbbell Bench Press: 3 sets × 10–12 repetitions
    ■ Alternative: Push-Ups on Knees (only go down as far as your mobility will allow)
  ○ Diamond Push-Ups: 3 sets × 10–12 repetitions
    ■ Alternative: Close-Grip Wall Push-Ups
  ○ Dumbbell Chest Flies (go light and pretend to “hug a tree”): 3 sets × 10–12 repetitions
    ■ Alternative: Wide-Grip Wall Push-Ups
  ○ Jack Knives: 2 sets × 20 repetitions
    ■ Alternative: Standard Crunches
- Cardio Workout
  ○ 30-min selected cardio exercise at high-intensity intervals: These intervals will be comprised of 1 min easy (RPE Scale: 2–3) and 1 min hard (RPE Scale: 7–8) and will be repeated until time is up.

Day 7:

- Strength Workout
  ○ Upright Rows: 3 sets × 10–12 repetitions
    ■ Alternative: Forward Arm Raises
  ○ One-Arm Rows (pretend you are “starting a lawnmower”): 3 sets × 10–12 repetitions
    ■ Alternative: Single Arm Circles
  ○ Lat Pulldowns: 3 sets × 10–12 repetitions
    ■ Alternative: Seated Reaching Exercise (count every other side)
  ○ Seated Cable Row: 2 sets × 10–12 repetitions
    ■ Alternative: Single Arm Circles
  ○ Sit-ups: 2 sets × 25 repetitions
    ■ Alternative: Standard Crunches
- Cardio Workout
  ○ 30-min selected cardio exercise at moderate pace: RPE Scale: 4–6

Day 8:

- Cardio Workout ONLY
  ○ 60-min selected cardio at light/moderate pace: RPE Scale: 3–6

Level 2. Exercise Made Easy: Intermediate Programming

Month 1: Endurance Phase

For each strength exercise, the endurance phase of this program will include higher numbers of repetitions (10–12 repetitions for each set) for each exercise with lower weight. You should rest for 60 s to 1.5 min between each strength exercise. For each cardio workout, feel free to select your favorite cardio exercise. Some cardio exercise options include but are not limited to walking, jogging, running, biking, swimming, stairclimbing, rowing, elliptical exercise, etc.

### Appendix A.3. Week 1

Day 1:

- Strength Workout
  ○ Dumbbell Lunges (count every other leg): 3 sets × 10–12 repetitions
    ■ Alternative: Walking Lunges without Dumbbells (still count every other leg)
  ○ Hamstring Curls: 3 sets × 10–12 repetitions
    ■ Alternative: Glute Kickbacks
  ○ Calf Raises: 3 sets × 50 repetitions
    ■ Alternative: Seated Heel Lifts
  ○ Bicycle Crunches: 2 sets × 50 revolutions
    ■ Alternative: Standard Crunches
- Cardio Workout
  ○ 30-min selected cardio exercise at moderate pace: RPE Scale: 4–6

Day 2:

- Cardio Workout Only
  ○ 30-min selected cardio at light/moderate pace: RPE Scale: 3–6

Day 3:

- Strength Workout
  ○ Dumbbell Bench Press: 3 sets × 10–12 repetitions
    ■ Alternative: Push-Ups on Knees or on Toes (only go as far down as your mobility will allow)
  ○ Tricep Kickbacks: 3 sets × 10–12 repetitions
    ■ Alternative: Tricep Dips on Chair (only go as far down as your mobility will allow)
  ○ Dumbbell Chest Flies (go light and pretend to “hug a tree”): 3 sets × 10–12 repetitions
    ■ Alternative: Wide-Grip Wall Push-Ups
  ○ Bicycle Crunches: 2 sets × 50 revolutions
    ■ Alternative: Sit-Ups
- Cardio Workout
  ○ 30-min selected cardio exercise at high-intensity intervals: These intervals will be comprised of 1 min easy (RPE Scale: 2–3) and 1 min hard (RPE Scale: 7–8) and will be repeated until time is up.

Day 4:

- Cardio Workout Only
  ○ 30-min selected cardio at light/moderate pace: RPE Scale: 3–6

Day 5:

- Strength Workout
  ○ Upright Rows: 3 sets × 10–12 repetitions
    ■ Alternative: Forward Arm Raises
  ○ One-Arm Rows (pretend you are “starting a lawnmower”): 3 sets × 10–12 repetitions
    ■ Alternative: Single Arm Circles
  ○ Lat Pulldowns: 3 sets × 10–12 repetitions
    ■ Alternative: Seated Reaching Exercise (count every other side)
  ○ Dumbbell Shrugs: 2 sets × 10–12 repetitions
    ■ Alternative: Shoulders to Ears
  ○ Sit-ups: 2 sets × 25 repetitions
    ■ Alternative: Bicycle Crunches or Standard Crunches
- Cardio Workout
  ○ 30-min selected cardio exercise at moderate pace: RPE Scale: 4–6

### Appendix A.4. Week 2

Day 6:

- Strength Workout
  ○ Goblet Squats with Dumbbell: 3 sets × 10–12 repetitions
    ■ Alternative: Air Squats
  ○ Hamstring Curls: 3 sets × 10–12 repetitions
    ■ Alternative: Glute Kickbacks
  ○ Split Jumps: 3 sets × 16 repetitions
    ■ Alternative: Walking Lunges without Dumbbells (count every other leg)
  ○ Leg Raises: 2 sets × 25 raises
    ■ Alternative: Standing Leg Raises (count every other leg)
- Cardio Workout
  ○ 30-min selected cardio exercise at moderate pace: RPE Scale: 4–6

Day 7:

- Cardio Workout Only
  ○ 30-min selected cardio at light/moderate pace: RPE Scale: 3–6

Day 8:

- Strength Workout
  ○ Incline Dumbbell Bench Press: 3 sets × 10–12 repetitions
    ■ Alternative: Push-Ups on Knees or Toes (only go as far down as your mobility will allow)
  ○ Diamond Push-Ups: 3 sets × 10–12 repetitions
    ■ Alternative: Close-Grip Wall Push-Ups
  ○ Dumbbell Chest Flies (go light and pretend to “hug a tree”): 3 sets × 10–12 repetitions
    ■ Alternative: Wide-Grip Wall Push-Ups
  ○ Jack Knives: 2 sets × 20 repetitions
    ■ Alternative: Sit-Ups or Standard Crunches
- Cardio Workout
  ○ 30-min selected cardio exercise at high-intensity intervals: These intervals will be comprised of 1 min easy (RPE Scale: 2–3) and 1 min hard (RPE Scale: 7–8) and will be repeated until time is up.

Day 9:

- Cardio Workout Only
  ○ 30-min selected cardio at light/moderate pace: RPE Scale: 3–6

Day 10:

- Strength Workout
  ○ Upright Rows: 3 sets × 10–12 repetitions
    ■ Alternative: Forward Arm Raises
  ○ Back Extension: 3 sets × 10–12 repetitions
    ■ Alternative: Good Morning Exercise (keep back straight through range of motion)
  ○ Lat Pulldowns: 3 sets × 10–12 repetitions
    ■ Alternative: Seated Reaching Exercise
  ○ Seated Cable Row: 2 sets × 10–12 repetitions
    ■ Alternative: Single Arm Circles
  ○ Sit-ups: 2 sets × 25 repetitions
    ■ Alternative: Bicycle Crunches or Standard Crunches
- Cardio Workout
  ○ 30-min selected cardio exercise at moderate pace: RPE Scale: 4–6
